# A Unique Presentation of a Thoracic Conundrum: Tracheobronchial Rests in the Esophagus

**DOI:** 10.7759/cureus.39096

**Published:** 2023-05-16

**Authors:** Abhijith Lakshman, Lawrence D'Cruze, Archana Balasubramaniam, Susruthan M, Periyasamy T

**Affiliations:** 1 Department of Pathology and Laboratory Medicine, Sri Ramachandra Institute of Higher Education and Research, Chennai, IND; 2 Department of Cardiothoracic Surgery, Sri Ramachandra Institute of Higher Education and Research, Chennai, IND

**Keywords:** rests, esophageal stenosis, heterotopia, congenital, choristoma, tracheobronchial

## Abstract

Tracheobronchial rests are a rare congenital anomaly where ectopic respiratory tract elements may be found in an abnormal site, such as within the esophageal wall. We present a case of a late presentation of an esophageal intramural tracheobronchial rest with complaints of pain in the left chest wall, vomiting, and loss of appetite for one month. The chest X-ray and mammogram were both normal, but an endoscopy could not be performed due to luminal narrowing. A CT scan shows a well-defined, round, non-enhancing hypodense lesion measuring 2.6 x 2.7 cm in the middle one-third of the esophagus.

Upon resection, histopathological examination revealed fragments of tissue lined by pseudostratified ciliated columnar epithelium with respiratory mucinous glands admixed with pools of mucin and underlying strands of skeletal muscle. The subepithelium contains esophageal submucosal glands, which confirm the esophageal origin of the choristoma.

The usual presentation is congenital esophageal stenosis at birth with over half of these cases being attributed to tracheobronchial rests. Presentation beyond adolescence is even rarer with a relatively benign course and favorable prognosis. Clinical, radiological, and pathological correlation as well as a high index of suspicion are important to avoid misdiagnosis and to institute optimal treatment.

## Introduction

Tracheobronchial rests are a rare congenital anomaly, with the first case being described in 1957, and are currently estimated to have an incidence of about 1 in 50,000-100,000 live births and only about 200 documented cases [1]. Tracheobronchial rests are choristomas, which are defined as a proliferation of histologically normal tissue in an abnormal site. These may form either due to the sequestration of tracheobronchial mesenchymal cells into the esophageal wall or improper division of the tracheobronchial bud during embryological development [2-5]. Most cases usually present early in life as congenital esophageal stenosis (CES) with fewer than 10% of these cases remaining asymptomatic beyond adolescence. More severe cases present with esophageal achalasia, which requires medical intervention early on. We present a rare case of an esophageal intramural tracheobronchial rest presenting with late esophageal stenosis, with differentials of an esophageal leiomyoma and fibroadenoma. This article was previously presented as a poster at the 2022 Diagnostic Challenges and Updates on Gastrointestinal and Hepatobiliary Neoplasms Conference on June 18, 2022.

## Case presentation

**Figure 1:**
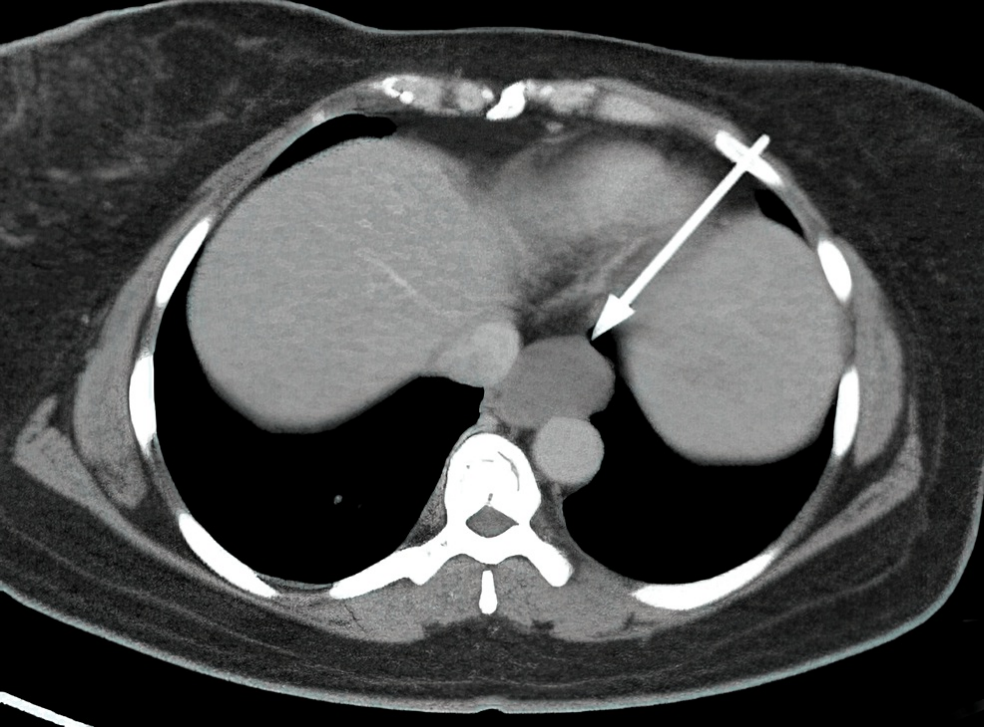
CT lung (transverse view) showing an esophageal mass (white arrow) A well-defined, round, non-enhancing hypodense lesion measuring 2.6 x 2.7 cm in the para-esophageal region on the left side from the middle one-third of the esophagus

A 46-year-old female presented to the Department of Cardiothoracic Vascular Surgery with complaints of pain in the left chest wall and heaviness of the left breast for 10 years. She also reported vomiting and a loss of appetite for one month. Initially, a breast lesion was suspected in view of chest pain and heaviness for 10 years and was repeatedly evaluated at other centers with no definitive diagnosis. However, the initial radiological work-up with a chest X-ray and mammogram was normal. Following this, an endoscopy was attempted but could not be performed due to severe luminal narrowing in the middle one-third of the esophagus. A CT lung examination revealed a lesion measuring 2.6 x 2.7 cm in the wall of the esophagus from the middle one-third segment, extending into the para-esophageal region on the left side (Figure 1). A well-defined fat plane between the lesion and the aorta was maintained.

**Figure 2:**
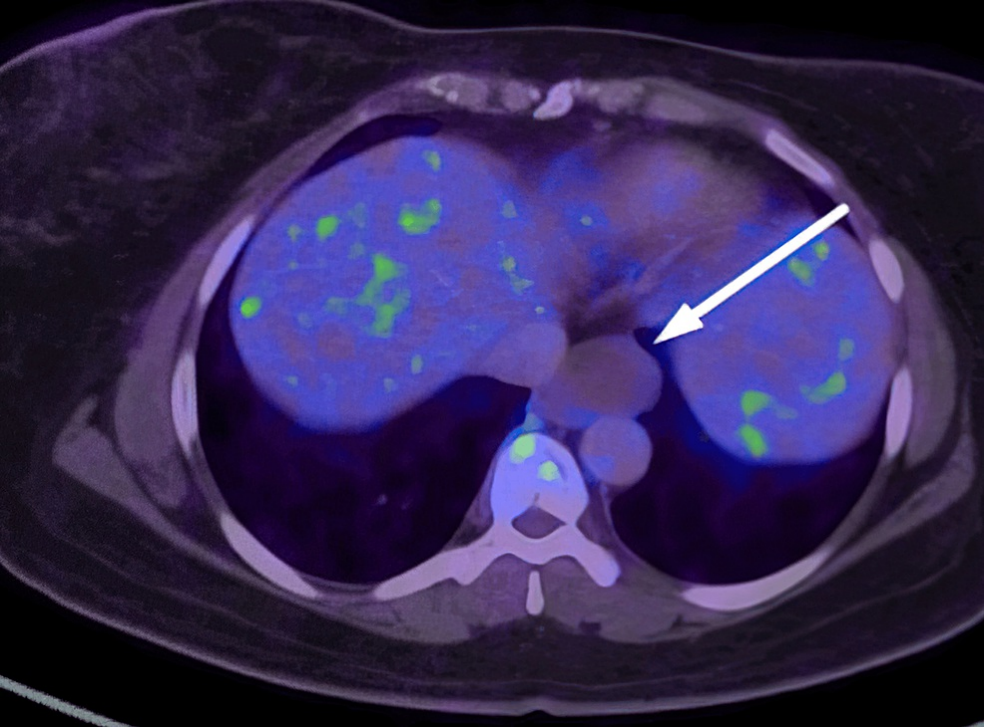
Non-FDG avid non-enhancing lesion (40 x 27 x 35 mm, SUV max 1.05) seen along the left lateral wall of the distal esophagus (white arrow)

A non-FDG avid non-enhancing lesion measuring 4 x 2.7 x 3.5 cm was also seen on PET-CT along the left lateral wall of the distal esophagus (Figure 2).

**Figure 3:**
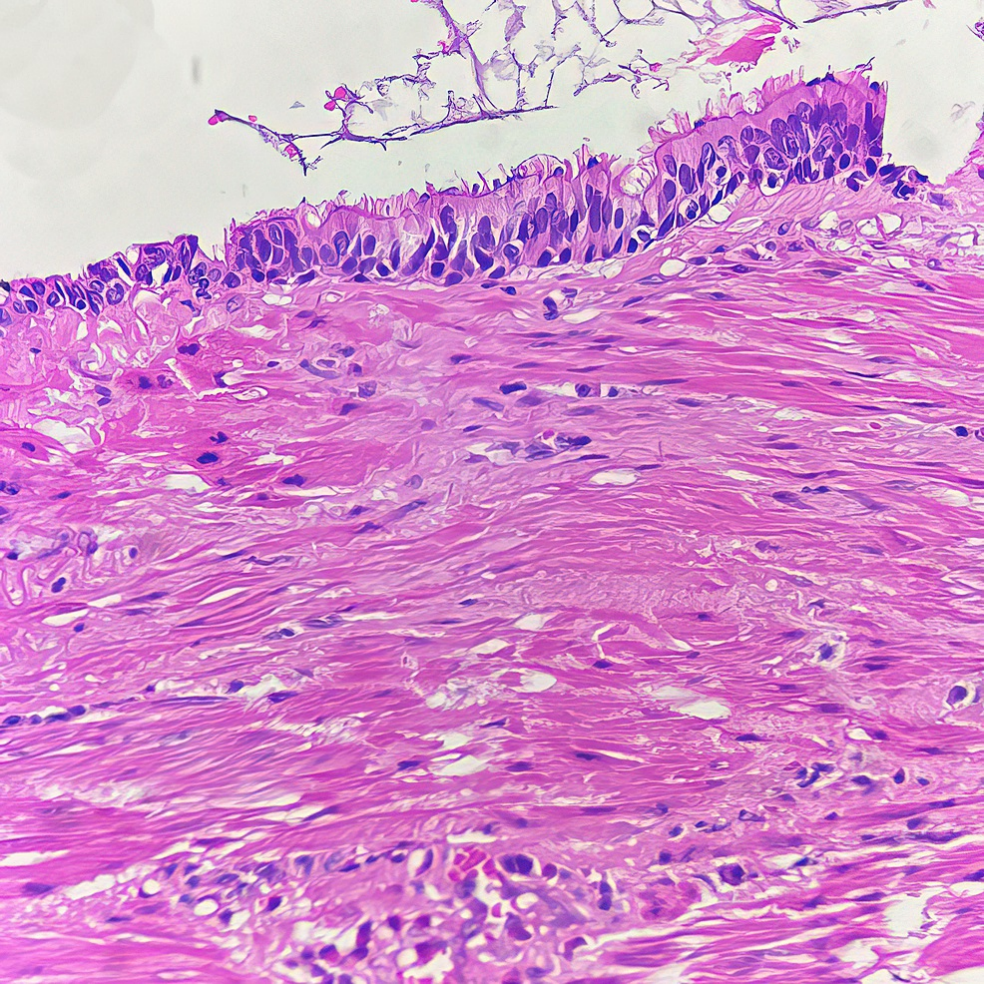
Respiratory epithelium with underlying bundles of skeletal muscle (H&E stain at 400x magnification)

These features were clinically suggestive of esophageal leiomyoma. Subsequent hematological tests and renal and liver function tests were normal. The lesion was endoscopically removed via a mucosal incision and enucleation of the mass under general anesthesia. The patient responded well to the surgery and was hemodynamically stable post-operatively. The specimen was received in toto for histopathological evaluation, measuring 2 x 1.5 x 1 cm, with a grey-white to grey-brown, irregular external surface and a grey-yellow, friable internal surface. Histopathological examination revealed fragments of tissue lined by pseudostratified ciliated columnar (respiratory) epithelium lying on bundles of striated skeletal muscle (Figure 3).

**Figure 4:**
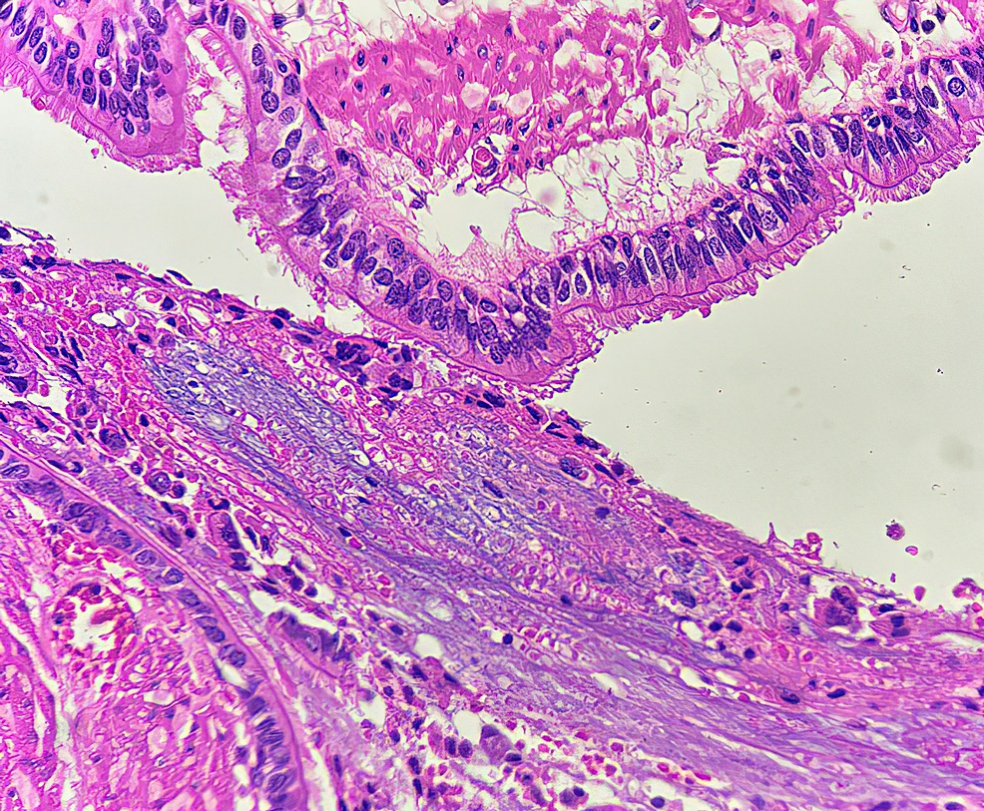
Respiratory epithelium and an adjacent pool of extracellular mucin containing numerous neutrophils and eosinophils (H&E stain at 400x magnification)

Numerous extracellular pools of mucin were also seen with admixed inflammatory cells, chiefly composed of neutrophils, eosinophils, and lymphocytes (Figure 4).

**Figure 5:**
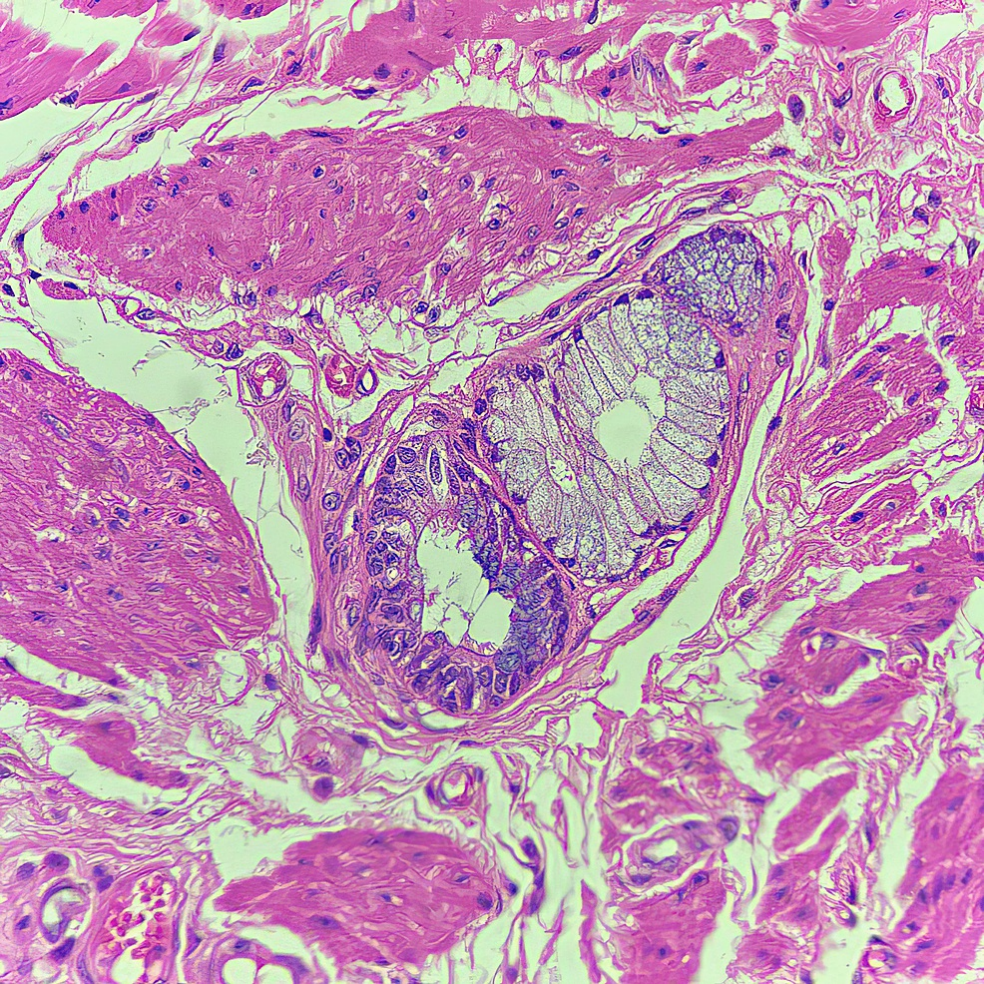
Subepithelium of the muscular fragment with an underlying submucosal gland, indicative of an esophageal origin (H&E stain at 400x magnification)

The underlying subepithelium also contains submucosal glands, which are suggestive of an esophageal origin (Figure 5).

An adjacent lymph node which was also excised along with the mass revealed changes secondary to reactive lymphadenitis. These findings confirm the presence of heterotopic elements within the esophagus, and a final diagnosis of esophageal intramural tracheobronchial rests was made.

## Discussion

Tracheobronchial choristomas are collections of heterotopic tissue present within the esophagus which contain various elements of the respiratory tract such as respiratory epithelium, bronchial glands, cartilage, and lymphatic tissue in the submucosa or muscularis propria and occur in the thoracic portion of the esophagus as seen in this case [6].

During embryological development, the trachea and esophagus share a common origin from the tracheobronchial bud which would further divide and develop to form these structures. The tracheobronchial groove, which lies anteriorly, gives rise to the respiratory tract and divides along the coronal plane. Jabari et al. described two predominant theories of division, the aforementioned "septation model" which is more widely accepted, as well as a "tracheal outpouch" model of development [7-8]. Separation usually occurs early in development, at 28-37 days after fertilization. The mid-esophageal location of the heterotopia may also be explained by the fact that the esophagus grows inferiorly at a faster rate, hence, coinciding with the upper and middle thirds of the esophagus at the time of division. The other postulate is localized metaplasia of mesenchymal cells in the wall of the esophagus.

The usual presentation is CES at birth with an incidence of 1 in 25,000 to 50,000 live births, 54% of these being attributed to tracheobronchial rests with an effective incidence of 1 in 50,000-100,000 live births [9-11]. Fewer than 10% of these cases present beyond adolescence. Other causes include fibromuscular thickening, esophageal cysts, and membranous webs, all of which are linked by a similar etiopathogenic link [12]. These findings are usually attributed to the degree of mechanical obstruction, which varies according to the size of the heterotopia. While larger rests present early in life due to extensive narrowing of the upper aerodigestive tract, smaller lesions, such as in this case, may only produce mild symptoms and would, hence, go unnoticed unless extensively evaluated. Lesions smaller than 3 cm may simply cause disruptions in the regular peristaltic movement of the esophagus or may remain occult, resulting in incidental detection [13]. Additionally, the lack of cartilaginous and lymphoepithelial elements may further contribute to the late presentation in this case, as the other glandular and lining epithelium that are present are insufficient to cause significant amounts of stenosis and, hence, have produced late symptoms. Radiologically, a hypoechoic mass may be seen beyond the muscularis propria. Tracheobronchial heterotopias are usually isolated lesions, and there is little data with reference to syndromic associations. However, recent studies have shown an association with various other malformations such as duodenal atresia, trisomy 21, tracheomalacia, and esophageal hiatal hernia [14].

The diagnosis is made by histopathological examination, which confirms the presence of respiratory components. Clinically, other intramural esophageal tumors may be close differentials, such as leiomyomas and gastrointestinal stromal tumors (GIST), which were ruled out with extensive sampling and thorough histopathological evaluation. Surgical intervention typically involves segmental resection or dilatation, though the former is preferred as dilatation usually produces unsatisfactory results, with an increased risk of recurrence and perforation. The lesion may be resected, such as in our case, where the residual thickness of the esophagus remains unaltered. Rests that involve a larger portion of the body and cause stenosis, on the other hand, may necessitate resection and anastomosis [5-8].

## Conclusions

Tracheobronchial rests are rare congenital lesions that can have a varied presentation depending on the size, with larger and more stenotic rests presenting early on in life as CES. The presentation of such choristomas is exceedingly rare in adulthood with non-specific symptoms and requires extensive evaluation for further management. While there is no known direct syndromic associations or genetic mutations, diagnosis of a tracheobronchial rest should be followed by screening for any other gastrointestinal malformations, as some case reports do mention the coexistence of such lesions.

Common radiological differentials include other esophageal intramural tumors such as leiomyoma and GIST, all of which may be differentiated microscopically. Surgical intervention typically involves segmental resection or, infrequently, dilatation. A high index of clinical suspicion, as in this case, as well as radiological and pathological correlation, is essential for avoiding misdiagnosis and initiating appropriate treatment.
